# Supplementary material for: Rapid genotyping of porcine reproductive and respiratory syndrome virus (PRRSV) using MinION nanopore sequencing
Source: PLoS One. 2023 May 23;18(5):e0282767. doi: 10.1371/journal.pone.0282767 (PMC10205005; doi:10.1371/journal.pone.0282767)
Supplement: S1 Table — (DOCX) [file pone.0282767.s001.docx]

| Sample ID | Collection State | Sample type | Ct value | WGS GenBank accession number | AmpliSeq GenBank accession number |
| --- | --- | --- | --- | --- | --- |
| USA/IL2020001051/2020 | IL | Oral fluid | 19.7 | MW592708 | MW810506 |
| USA/IA2019093435/2019 | IA | Oral fluid | 23.2 |  | MW810550 |
| USA/NE2019076796/2019 | NE | Oral fluid | 24 |  | MW810551 |
| USA/IN2019073234/2019 | IN | Oral fluid | 24.8 | MW592726 | MW810507 |
| USA/UT2019073234/2019 | UT | Oral fluid | 25 |  | MW810559 |
| USA/OK2019069536/2019 | OK | Oral fluid | 25.1 |  | MW810552 |
| USA/OH2019073156-3/2019 | OH | Oral fluid | 25.3 |  | MW810572 |
| USA/MN2019070883/2019 | MN | Oral fluid | 25.4 | MW592732 | MW810561 |
| USA/IA2019070459/2019 | IA | Oral fluid | 25.4 |  | MW810579 |
| USA/IA2019068364/2019 | IA | Oral fluid | 25.5 |  | MW810508 |
| USA/IA2019071849/2019 | IA | Oral fluid | 25.5 |  | MW810548 |
| USA/OK2019076371/2019 | OK | Oral fluid | 25.5 |  | MW810562 |
| USA/OH2019073156/2019 | OH | Oral fluid | 25.6 | MW592728 | MW810576 |
| USA/IA2019070924/2019 | IA | Oral fluid | 25.7 |  | MW810553 |
| USA/IA2019071849-12/2019 | IA | Oral fluid | 25.7 | MW592731 | MW810549 |
| USA/NC2019071083/2019 | NC | Oral fluid | 26.4 |  | MW810509 |
| USA/IA2019070117/2019 | IA | Oral fluid | 26.4 |  | MW810573 |
| USA/IA2019074174/2019 | IA | Oral fluid | 26.5 |  | MW810582 |
| USA/AZ2019070453/2019 | AZ | Oral fluid | 26.7 |  | MW810577 |
| USA/IA2019075441/2019 | IA | Oral fluid | 27 |  | MW810540 |
| USA/IA2019075047/2019 | IA | Oral fluid | 27.3 |  | MW810580 |
| USA/UT2019073234-36/2019 | UT | Oral fluid | 27.8 |  | MW810585 |
| USA/OH2019075797/2019 | OH | Oral fluid | 28 | MW592723 | MW810542 |
| USA/IA2019072113/2019 | IA | Oral fluid | 28.2 |  | MW810541 |
| USA/NC2020002293/2020 | NC | Oral fluid | 28.8 | MW592718 | MW810538 |
| USA/NC2019071648/2019 | NC | Oral fluid | 28.9 |  | MW810522 |
| USA/IA2019068763/2019 | IA | Oral fluid | 29 |  | MW810578 |
| USA/IA2019069365/2019 | IA | Oral fluid | 29.4 |  | MW810574 |
| USA/IL2019096198/2019 | IL | Proc.fluid | 17 |  | MW810544 |
| USA/IA2020002279/2020 | IA | Proc.fluid | 19.2 | MW592709 | MW810523 |
| USA/2019074725/2019 | NC | Proc.fluid | 20.4 |  | MW810537 |
| USA/NC2019093091/2019 | NC | Proc.fluid | 20.4 | MW592716 | MW810525 |
| USA/IA2019074336/2019 | IA | Proc.fluid | 21.2 | MW592719 | MW810534 |
| USA/IA2019071643/2019 | IA | Proc.fluid | 21.3 | MW592720 | MW810535 |
| USA/IA2019091323/2019 | IA | Proc.fluid | 21.5 | MW592734 | MW810568 |
| USA/NE2019092592/2019 | NE | Proc.fluid | 21.6 | MW592715 | MW810528 |
| USA/MN2019092568/2019 | MN | Proc.fluid | 21.9 | MW592729 | MW810527 |
| USA/MN2019075338/2019 | MN | Proc.fluid | 22 | MW592730 | MW810545 |
| USA/NC2019095720/2019 | NC | Proc.fluid | 22 | MW592717 | MW810530 |
| 2019092601/2019 | Unknown | Proc.fluid | 22.2 | MW592724 | MW810543 |
| USA/IA2019095692/2019 | IA | Proc.fluid | 22.3 | MW592721 | MW810536 |
| USA/IA2019072096/2019 | IA | Proc.fluid | 22.6 | MW592737 | MW810526 |
| USA/IA2019072150/2019 | IA | Proc.fluid | 22.9 |  | MW810566 |
| USA/IA2019076844/2019 | IA | Proc.fluid | 23.5 | MW592736 | MW810567 |
| USA/MO2019072088/2019 | MO | Proc.fluid | 24.3 | MW592725 | MW810533 |
| USA/NE2019074755/2019 | NE | Proc.fluid | 25.3 | MW592739 | MW810555 |
| USA/NE2019072008/2019 | NE | Proc.fluid | 25.7 |  | MW810557 |
| USA/IA2019075406/2019 | IA | Proc.fluid | 26 |  | MW810570 |
| USA/KS2019095307/2019 | KS | Proc.fluid | 26.6 |  | MW810564 |
| 2020000666/2020 | Unknown | Proc.fluid | 26.6 |  | MW810546 |
| USA/IA2020000194/2020 | IA | Proc.fluid | 27.2 |  | MW810556 |
| 2019074509/2019 | Unknown | Proc.fluid | 27.3 |  | MW810560 |
| 2019074516/2019 | Unknown | Proc.fluid | 27.5 |  | MW810521 |
| USA/IA2019094759/2019 | IA | Proc.fluid | 27.5 |  | MW810531 |
| USA/IA2019073213/2019 | IA | Proc.fluid | 27.9 |  | MW810510 |
| USA/NC2020001837/2020 | NC | Proc.fluid | 28.1 |  | MW810539 |
| USA/NE2019074758/2019 | NE | Proc.fluid | 28.9 |  | MW810529 |
| USA/OK2019072118/2019 | OK | Proc.fluid | 29.3 |  | MW810558 |
| USA/NE2019072006/2019 | NE | Proc.fluid | 29.4 |  | MW810554 |
| USA/IA2019075048/2019 | IA | Proc.fluid | 29.8 |  | MW810586 |
| USA/NE2019072577/2019 | NE | Proc.fluid | 29.9 |  | MW810524 |
| USA/NC2020000873/2020 | NC | Proc.fluid | 30.6 |  | MW810583 |
| USA/IA2019095060/2019 | IA | Serum | 15 | MW592733 | MW810565 |
| USA/IA2019070359/2019 | IA | Serum | 15.1 | MW592710 | MW810511 |
| USA/IA2019069719/2019 | IA | Serum | 17.4 | MW592712 | MW810512 |
| USA/IA2019068844/2019 | IA | Serum | 17.9 | MW592711 | MW810518 |
| USA/TX2019095629/2019 | TX | Serum | 19.3 | MW592738 | MW810563 |
| USA/IA2019071609/2019 | IA | Serum | 21.5 | MW592714 | MW810513 |
| USA/IA2019070358/2019 | IA | Serum | 22 | MW592713 | MW810514 |
| USA/IA2019094301-1/2019 | IA | Serum | 22 | MW592735 | MW810569 |
| USA/IA2019068844-46/2019 | IA | Serum | 23 |  | MW810575 |
| USA/IA2019071609-88/2019 | IA | Serum | 24.1 |  | MW810584 |
| 2019093766/2019 | Unknown | Serum | 26.1 |  | MW810520 |
| USA/AZ2019095301/2019 | AZ | Serum | 26.4 |  | MW810571 |
| USA/IA2019098151/2019 | IA | Serum | 27.2 | MW592722 | MW810519 |
| USA/IA2019095454/2019 | IA | Serum | 27.3 | MW592727 | MW810581 |
| 2020001553/2020 | Unknown | Serum | 27.7 |  | MW810517 |
| USA/IL2019098027/2019 | IL | Serum | 28.5 |  | MW810532 |
| 2020001567/2020 | Unknown | Serum | 30 |  | MW810547 |
| USA/IA2020002280/2020 | IA | Serum | 30.2 |  | MW810515 |
| 2020001554/2020 | Unknown | Serum | 30.8 |  | MW810516 |
| 2021073181 | Unknown | Lung | 22.2 | OQ361816 |  |
| 2021085554 | Unknown | Lung | 15.76 | OQ361815 |  |
| 2021039141 lung 1 | Unknown | Lung | 29.82 | OQ361807 |  |
| 2021039141 lung 2 | Unknown | Lung | 27.19 | OQ361808 |  |
| 2021042219 lung 3 | Unknown | Lung | 17.46 | OQ361809 |  |
| 2021042219 lung 4 | Unknown | Lung | 17.75 | OQ361811 |  |
| 2021056440 lung 1 | Unknown | Lung | 20.55 | OQ361812 |  |
| 2021066033 lung GA | Unknown | Lung | 19.78 | OQ361822 |  |
| 2021069845 lung 2 | Unknown | Lung | 25.47 | OQ361824 |  |
| 2021073186 lung GA | Unknown | Lung | 20.38 | OQ361823 |  |
| 2021079016 lung 1 | Unknown | Lung | 23.3 | OQ361817 |  |
| 2021079016 lung 2 | Unknown | Lung | 25.01 | OQ361818 |  |
| 2021090462 lung 1 | Unknown | Lung | 21.06 | OQ361819 |  |
| 2021090462 lung 2 | Unknown | Lung | 19.59 | OQ361820 |  |
| 2021090462 lung 3 | Unknown | Lung | 28.87 | OQ361821 |  |
| 2021092855 lung 1 | Unknown | Lung | 22.17 | OQ361813 |  |
| 2021092855 lung 2 | Unknown | Lung | 23.14 | OQ361814 |  |
| 2021093514 lung GA | Unknown | Lung | 18.7 | OQ361810 |  |
