# Supplementary material for: Rapid genotyping of porcine reproductive and respiratory syndrome virus (PRRSV) using MinION nanopore sequencing
Source: PLoS One. 2023 May 23;18(5):e0282767. doi: 10.1371/journal.pone.0282767 (PMC10205005; doi:10.1371/journal.pone.0282767)
Supplement: S2 Table — (DOCX) [file pone.0282767.s002.docx]

1)

medaka_haploid_variant -i samplename.fastq -r reference.fasta -o outputfolder -m r941_min_hac_variant_g507 -s -t 12

2)

for i in $(ls *.vcf); do echo "$i $(bcftools query -f'%CHROM\t%POS0\t%END\n' $i* > ${i}_variants.bed)"; done

3)

for i in $(ls *.bam); do echo "$i $(bedtools genomecov -bga -ibam $i* | awk '$4 < 20' > ${i}_low_coverage_sites.bed)"; done

4)

bedtools subtract -a *samplename*_low_coverage_sites.bed -b *samplename*_variants.bed > *samplename* mask.bed

5)

for i in $(ls *.vcf); do echo "$i $(bgzip $i)"; done

6)

for i in $(ls *.vcf.gz); do echo "$i $(tabix $i)"; done

7)

bcftools consensus -p *name* -f *reference*.fasta --mark-del '-' -m *name*_mask.bed -i '%QUAL >= 20' *name*.vcf.gz | sed "/^>/s/*name*.*/*name*/" > *name*.bcftools.consensus.fa
