# Supplementary material for: Rapid genotyping of porcine reproductive and respiratory syndrome virus (PRRSV) using MinION nanopore sequencing
Source: PLoS One. 2023 May 23;18(5):e0282767. doi: 10.1371/journal.pone.0282767 (PMC10205005; doi:10.1371/journal.pone.0282767)
Supplement: S3 Table — Twenty-four clinical samples were randomly selected by sample type and Ct range to provide detailed results of sequencing accuracy. (DOCX) [file pone.0282767.s003.docx]

| **Sample type** | **Sample ID** | **PRRSV qPCR Ct** | **Total n. of reads** | **N. of mapped PRRSV reads / % of total** | **Top BLAST match** | **Identity to top BLAST match** | **Genome Coverage >20X** |
| --- | --- | --- | --- | --- | --- | --- | --- |
| Lung | 2021085554 | 15.76 | 70200 | 57636 / 82.10% | OL963969.1 | 98.75 | 99.65 |
|  | 2021093514_lung_GA | 18.7 | 66019 | 60084 / 91.01% | OL963979.1 | 98.81 | 99.66 |
|  | 2021073186_lung_GA | 20.38 | 74425 | 51125 / 68.69% | MF326990.1 | 96.1 | 91.91 |
|  | 2021092855_lung_2 | 23.14 | 52524 | 43420 / 82.67% | OL963967.1 | 98.81 | 99.64 |
|  | 2021079016_lung_2 | 25.01 | 69460 | 43486 / 62.61% | OL963979.1 | 98.44 | 98.55 |
|  | 2021039141_lung_1 | 29.82 | 37674 | 26037 / 69.11% | OL963961.1 | 92.89 | 99.65 |
| Oral fluid | USA/IL2020001051/2020 | 19.7 | 171735 | 148778 / 86.63% | MF326985.1 | 96.28 | 99.42 |
|  | USA/IA2019093435/2019 | 23.2 | 177581 | 61603 / 34.69% | MN073137.1 | 98.03 | 99.31 |
|  | USA/NE2019076796/2019 | 24 | 201854 | 80373 / 39.82% | MN073139.1 | 94.43 | 91.64 |
|  | USA/UT2019073234/2019 | 25 | 89212 | 36306 / 40.70% | MN073159.1 | 99.13 | 75.40 |
|  | USA/IA2019075441/2019 | 27 | 88893 | 47147 / 53.04% | OL963962.1 | 97.41 | 93.66 |
|  | USA/IA2019069365/2019 | 29.4 | 71549 | 15403 / 21.53% | MN073108.1 | 96.74 | 91.71 |
| Processing fluid | USA/IL2019096198/2019 | 17 | 210625 | 158946 / 75.46% | MN073092.1 | 95.33 | 98.54 |
|  | USA/IA2020002279/2020 | 19.2 | 93802 | 85805 / 91.47% | MF326985.1 | 97 | 99.66 |
|  | USA/IA2019074336/2019 | 21.2 | 140628 | 126926 / 90.26% | MN073081.1 | 98.92 | 99.40 |
|  | USA/MO2019072088/2019 | 24.3 | 119326 | 102372 / 85.79% | MN073101.1 | 98.76 | 99.65 |
|  | USA/NE2019072008/2019 | 25.7 | 28253 | 6264 / 22.17% | MN073133.1 | 99.47 | 76.96 |
|  | USA/NC2020000873/2020 | 30.6 | 86713 | 3225 / 3.72% | KF287141.1 | 99.62 | 44.94 |
| Serum | USA/IA2019095060/2019 | 15 | 238846 | 176710 / 73.98% | MN073108.1 | 94.55 | 99.95 |
|  | USA/IA2019068844/2019 | 17.9 | 136112 | 135810 / 99.78% | MN073092.1 | 99.79 | 99.65 |
|  | USA/IA2019071609/2019 | 21.5 | 88343 | 84729 / 95.91% | MN073092.1 | 99.73 | 99.65 |
|  | USA/IA2019071609-88/2019 | 24.1 | 108362 | 106506 / 98.29% | MN073092.1 | 99.81 | 99.65 |
|  | 2019093766/2019 | 26.1 | 19640 | 11093 / 56.48% | KT894735.1 | 89.95 | 78.26 |
|  | 2020001567/2020 | 30 | 28218 | 207 / 0.73% | MN073092.1 | 99.68 | 41.27 |
